# Gag Virus-like Particles Functionalized with SARS-CoV-2 Variants: Generation, Characterization and Recognition by COVID-19 Convalescent Patients’ Sera

**DOI:** 10.3390/vaccines11111641

**Published:** 2023-10-26

**Authors:** Arnau Boix-Besora, Francesc Gòdia, Laura Cervera

**Affiliations:** Grup d’Enginyeria de Bioprocessos i Biocatàlisi Aplicada ENG4BIO, Escola d’Enginyeria, Universitat Autònoma de Barcelona, Cerdanyola del Vallès, 08193 Barcelona, Spain

**Keywords:** SARS-CoV-2, variants, spike, virus-like particles, COVID-19, D614G, furin cleavage

## Abstract

The robustness, safety, versatility, and high immunogenicity of virus-like particles (VLPs) make them a promising approach for the generation of vaccines against a broad range of pathogens. VLPs are recombinant macromolecular structures that closely mimic the native conformation of viruses without carrying viral genetic material. Particularly, HIV-1 Gag-based VLPs are a suitable platform for the presentation of the SARS-CoV-2 Spike (S) protein on their surface. In this context, this work studies the effect of different rationally engineered mutations of the S protein to improve some of its characteristics. The studied variants harbored mutations such as proline substitutions for S stabilization, D614G from the early dominant pandemic form, the elimination of the S1/S2 furin cleavage site to improve S homogeneity, the suppression of a retention motif to favor its membrane localization, and cysteine substitutions to increase its immunogenicity and avoid potential undesired antibody-dependent enhancement (ADE) effects. The influence of the mutations on VLP expression was studied, as well as their immunogenic potential, by testing the recognition of the generated VLP variants by COVID-19 convalescent patients’ sera. The results of this work are conceived to give insights on the selection of S protein candidates for their use as immunogens and to showcase the potential of VLPs as carriers for antigen presentation.

## 1. Introduction

The coronavirus disease 2019 (COVID-19) pandemic caused by severe acute respiratory syndrome 2 (SARS-CoV-2) has had a brutal health and economic impact worldwide [1,2]. Vaccines constitute a major part of the solution to mitigate the expansion of this virus, its emerging variants, or the zoonotic threat posed by new coronaviruses with pandemic potential [3,4]. The approval of multiple commercial vaccines, their intensive manufacturing, and the deployment of the corresponding vaccination programs resulted in a reduction in transmissions, associated hospitalizations, and deaths, making it possible for the sanitary restrictions to come to an end [5]. However, none of them confer a full prophylactic protection for a long period of time, and new emerging variants are evolving with potential to escape the immune protective effect of the vaccinated population [6,7,8]. In the present landscape of vaccine development, DNA/RNA-based, viral vector-based, and VLP-based vaccines are generating substantial interest. Virus-like particles (VLPs) constitute a high-immunogenic, versatile, robust and safe approach with great potential as vaccine candidates [9,10]. They induce potent cellular and humoral responses, which makes the use of adjuvants optional, and can be pseudotyped to present different epitopes of interest per particle when used as a vaccine [11].

HIV-1 Gag based VLPs are particles of ~145 nm diameter that have been successfully functionalized to present SARS-CoV-2 and foot-and-mouth disease proteins [12,13]. Further, their thermostability and aggregation have been studied in order to assess their resistance at different storage conditions, concluding that Gag VLPs are stable for up to three months at 4 °C or −80 °C [14]. They are generated by the recombinant expression of the HIV-1 polyprotein, which accumulates at the membrane of the producer cells and buds from them, taking part of the cell’s plasmatic membrane as its lipidic envelope [15,16]. If the producer cell is simultaneously expressing other membrane proteins, those are incorporated into the surface of Gag VLPs [12]. This can be taken as an advantage to functionalize them with SARS-CoV-2 proteins.

For this purpose, mammalian platforms constitute a promising approach for the expression of enveloped VLPs due to their ability to assemble VLPs in the desired native viral antigenic configurations, and perform complex post-translational modifications (PTMs) [16,17,18]. Among these, the Human Embryonic Kidney (HEK) 293 cell line has been globally used for recombinant protein expression due to its good characterization, high transfection efficiency, product quality, and capability to grow in suspension in bioreactors in chemically-defined serum-free media [12,19].

**Figure 1:**
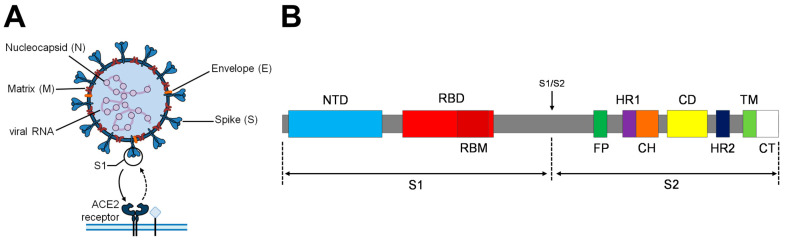
(**A**): SARS-CoV-2 virion scheme. M, N, E and S proteins are represented. S1 subunit of the S protein interacts with the host cell receptor membrane protein ACE2 to bind and promote viral internalization. (**B**): Schematic representation of the Spike protein. S1/S2 cleavage site is indicated with an arrow. Abbreviations: NTD, N-terminal domain; RBD, receptor-binding domain; RBM, receptor-binding motif; FP, fusion peptide; HR1 and HR2, heptad repeat 1 and 2; CH, central helix; CD, connector domain; TM, transmembrane domain; CT, cytoplasmic tail.

SARS-CoV-2 virus is formed by four structural proteins: matrix (M), envelope (E), nucleocapside (N), and Spike (S) (Figure 1A). The spike glycoprotein is present on the surface of the viral particles forming prominent homotrimers. It is a type I transmembrane fusion protein composed by 1273 amino acids (aa) divided into two subunits: S1 (1–685 aa) and S2 (686–1273 aa). S1 is constituted by the N-terminal domain (NTD) and the receptor-binding domain (RBD) (Figure 1B). The RBD receptor-binding motif (RBM) interacts and binds to the angiotensin-converting enzyme-2 (ACE-2) receptor present at the surface of some host cell types [20]. S2 is a less exposed S subunit responsible for the fusion between the host cell and viral membranes [21], whose domains are detailed in Figure 1B. After RBD interaction with the ACE-2 receptor, host cell protease cleavage induces large conformational changes, resulting in the exposure of the S2 fusion machinery allowing membrane fusion and viral entry [22]. Overall, this critical role of the spike in the life cycle of the viral infection makes it the primary target for the development of preventive therapies and vaccines [22,23]. Small mutations can affect its transmission, pathogenicity, and immunogenicity. For that reason, they need to be studied and considered for the advancement in SARS-CoV-2 vaccine development [20].

The incorporation of the S-protein at the surface of the Gag-based VLPs generates SARS-CoV-2 functionalized VLPs (S-VLPs) [12]. In this work, S was rationally engineered by modifying its nucleotide sequence to propose and study different S variants. The introduced mutations consist of stabilizing proline substitutions [24,25]; D614G as the early dominant pandemic form [26]; the substitution of three arginine codons in order to eliminate the S1/S2 polybasic cleavage site [22,27]; lysine and histidine substitutions at the C-terminal in order to eliminate the ER-Golgi intermediate compartment (ERGIC) retention dibasic motif [28]; and two cysteine substitutions to create a disulfide bond in order to avoid antibody-dependent enhancement (ADE) response [22], a phenomenon that can occur when the antibodies generated after immunization are not able to stop the infection and instead act as a “Trojan horse” facilitating pathogen cellular entry [29,30]. In this study, three S-VLP candidates harboring different S protein mutations were generated and their recognition by sera from COVID-19 patients was tested. This allowed its immunogenic potential to be determined and the more promising immunogen to be selected for further study as a potential vaccine candidate.

## 2. Materials and Methods

### 2.1. Cell Line, Media, and Culture Conditions

The serum-free suspension-adapted HEK293 cell line (HEK293SF-3F6) was used, kindly provided by Dr. Amine Kamen from the Biotechnology Research Institute at the National Research Council of Canada and McGill University (Montreal, Canada). This cell line was derived from a current good manufacturing practice (cGMP) master cell bank available for manufacturing of clinical material.

The medium used for HEK293 cellular growth was the chemically defined and free-from-animal-components HyCell TransFx-H from HyClone (GE Healthcare, Chicago, IL, USA), supplemented with 4 mM GlutaMAX (Gibco, Thermo Fisher Scientific, Waltham, MA, USA) and 0.1% Pluronic F-68 Non-ionic Surfactant (Gibco, Thermo Fisher Scientific, Waltham, MA, USA).

Suspension cell cultures were maintained routinely in exponential growth phase in 125 mL or 1 L disposable polycarbonate Erlenmeyer flasks with a vent cap (Corning, Tewksbury, MA, USA) in a LT-X Kuhner shaker (LT-X Kuhner, Birsfelden, Switzerland), shaking at 130 rpm, at 37 °C, 5% CO_2_, and 85% RH. Cell counts and viability determinations were performed using the NucleoCounter NC-3000 automatic cell counter (Chemometec, Lillerød, Denmark) following the manufacturer’s instructions.

### 2.2. Plasmids and Transfection

#### 2.2.1. Plasmid Expression Vectors

The pGag::eGFP plasmid codes for a codon-optimized Rev-independent HIV-1 Gag protein fused in frame to the enhanced GFP driven by the CMV enhancer and promoter. The plasmid from the NIH AIDS Reagent Program (Cat 11468) [31] was constructed by cloning the Gag sequence from pCMV55M1-10 [32] into the pEGFP-N1 plasmid (Clontech, Palo Alto, CA, USA).

The pSpike plasmid codes for a mammalian cell codon optimized nucleotide sequence of the spike protein of SARS-CoV-2 driven by the CAG enhancer and β-actin promoter. It was produced under HHSN272201400008C and obtained through BEI Resources, NIAID, NIH: Vector pCAGGS Containing the SARS-CoV-2, Wuhan-Hu-1 spike Glycoprotein Gene, NR-52310.

The pSpikemut2 plasmid codes for a mammalian cell codon optimized nucleotide sequence of the Spike protein of SARS-CoV-2 (Wuhan-Hu-1 spike Glycoprotein Gene, NR-52310) harboring K986P, V987P, S383C, D985C, D614G, and R682_R685delinsGSAS mutations. The expression is driven by the CAG enhancer and β-actin promoter. It was designed by A. Boix-Besora, and produced by Gene Synthesis & DNA Synthesis Services of GenScript (GenScript, Leiden, The Netherlands), derived from pSpike. Sequencing and restriction analysis were carried out to validate the construct.

The pSpikemut3 plasmid codes for a mammalian cell codon optimized nucleotide sequence of the Spike protein of SARS-CoV-2 (Wuhan-Hu-1 spike Glycoprotein Gene, NR-52310) harboring K986P, V987P, S383C, D985C, D614G, R682_R685delinsGSAS, and K1269A H1271A mutations. The expression is driven by the CAG enhancer and β-actin promoter. It was designed by A. Boix-Besora, and produced by Gene Synthesis & DNA Synthesis Services of GenScript (GenScript, Leiden, The Netherlands), derived from pSpike. Sequencing and restriction analysis were carried out to validate the construct.

pMock plasmid does not have any mammalian promoter or coding DNA sequence (CDS). It was constructed by the ligation of the pGag::eGFP backbone.

#### 2.2.2. Plasmid Amplification and Purification

Plasmids were amplified in *Escherichia coli* DH5α strain grown in LB medium (Conda, Madrid, Spain) supplemented with kanamycin (10 µg/mL, Sigma, St. Louis, MO, USA) or ampicillin (100 µg/mL, Sigma, St. Louis, MO, USA) depending on the *E. coli* antibiotic resistance present on each plasmid. Plasmid purification was carried out using the Endofree Plasmid Mega kit (Qiagen, Hilden, Germany) according to the manufacturer’s instructions.

#### 2.2.3. PEI-Mediated Transient Transfection

Exponentially growing HEK293 cells were passaged in 1 L polycarbonate Erlenmeyer flasks to reach a cell density of 2 × 10^6^ cells/mL at transfection time. A medium exchange was performed prior transfection by centrifugation of the cells at 300× *g* for 5 min. A total of 25 kDa linear polyethylenimine (PEI Max, PolySciences, Warrington, PA, USA) was used as transfection reagent. PEI-DNA complexes were formed under sterile conditions. Briefly, DNA was diluted in culture media (10% of the total volume of cell culture to be transfected) for a final total DNA concentration of 1 µg/mL and vortexed for 10 s. Then, polyethylenimine (PEI) was added for a final concentration of 2 µg/mL (a 2:1 PEI:DNA ratio (*w*/*w*)) and vortexed three times for 3 s. The mixture was incubated for 15 min at RT and then added to the culture.

### 2.3. Immunocytochemistry Staining for Flow Citometry and Confocal microscopy

For IF-ICC staining, cells were centrifuged 5 min at 300× *g* and rinsed with staining solution (1.5% (*v*/*v*) fetal bovine serum (FBS) 1X phosphate-buffered saline (PBS)) before primary antibody incubation for 20 min at 4 °C in the dark. After rinsing twice, cells were incubated with the corresponding secondary antibody for 20 min at 4 °C. After IF-ICC staining, fixation was performed using 2% (*v*/*v*) formaldehyde in PBS for 10 min at RT. Cells were resuspended in staining solution and stored at 4 °C prior to analysis.

Primary human anti-SARS-CoV-2 spike glycoprotein RBD domain antibody (ab272854, AbCam, Cambridge, UK) was diluted 1:1000. The secondary antibody used for flow cytometry analysis was an anti-human IgG (H + L) coupled with Cy™5, produced in donkey (709-175-149, Jackson ImmunoResearch, West Grove, PA, USA), diluted 1:400. All IF-ICC antibodies were diluted using staining solution.

The transfected cellular populations of previously stained cells were analyzed by flow cytometry using a BD FACS Canto flow cytometer (BD BioSciences, San Jose, CA, USA), at Servei de Cultius Cel·lulars, Producció d’Anticossos i Citometria (Universitat Autònoma de Barcelona, Bellaterra, Catalonia, Spain).

### 2.4. Confocal Microscopy

For confocal microscopy imaging, cells were treated and stained as described in the previous section. The secondary antibody used was an anti-human IgG (H + L) produced in goat coupled with Alexa Fluor 568, (#A-21090, Thermo Fisher Scientific, Waltham, MA, USA). It was diluted 1:400 in staining solution.

Prior to visualization, cells were treated with 0.1% (*v*/*v*) of Hoechst 33342 (Thermo Fisher Scientific, Waltham, MA, USA) in order to stain cellular nuclei. Samples were placed in 35 mm glass-bottomed Petri dishes with 14 mm microwells (MatTek Corporation, Ashland, MA, USA).

IF-ICC cells were imaged using a Leica TCS SP5 (Leica Microsystems, Wetzlar, Germany) confocal fluorescence microscope at Servei de Microscòpia de la facultat de Biocicències (Universitat Autònoma de Barcelona). The laser wavelengths used were (λ_ex_ 488 nm, λ_em_ 510 nm) for Gag::eGFP, (λ_ex_ 578 nm, λ_em_ 603 nm) for Alexa 568 and (λ_ex_ 353 nm, λ_em_ 453 nm) for Hoechst. No anti-fade solution was needed.

### 2.5. Transmission Electron Microscopy (TEM), Negative Staining

Transmission Electron Microscopy analyses were carried on at Servei de Microscòpia from Universitat Autònoma de Barcelona (Bellaterra, Catalonia, Spain). Sample visualization was performed in a JEOL 2011 transmission electron microscope (Jeol, Tokio, Japan) operating at an accelerating voltage of 200 kV. Electron micrographs were recorded with the Digital Micrograph software package GMS 3.3.1 (Gatan, Pleasanton, CA, USA). Images were recorded by a Gatan US4000 (Gatan, Pleasanton, CA, USA) cooled charge-coupled device (CCD) camera.

Negative staining was performed by means of the air-dried method. Briefly, an aliquot of purified VLPs was absorbed by flotation onto freshly glow-discharged 400-mesh carbon film copper grids (22-1MC040-100, MicrotoNano, Haarlem, The Netherlands). After standing for 1 min at RT, excess sample was drained off the grid carefully using Grade 1 Whatman filter paper (WHA1001325, Merck, Kenilworth, NJ, USA). Samples were then treated with 5 µL of uranyl acetate (2%) and incubated for 1 min at RT. The excess uranyl acetate was drained off as previously described.

### 2.6. Sucrose Cushion Purification

Culture harvests were performed at 72 hpt and centrifuged 10,000× *g* for 10 min. The supernatants containing VLPs were placed on a 30% (*w*/*v*) sucrose cushion for ultracentrifugation at 31,000 rpm for 2 h at 4 °C. The supernatant was carefully discarded, and pellets containing the VLPs were resuspended in PBS.

### 2.7. Nanoparticle Tracking Analysis

NTA-based Gag::eGFP VLP quantification and characterization was performed using a NanoSight^®^NS300 (Nanosight Ltd., Amesbury, UK) equipped with a blue filter module (488 nm) and a neutral filter at the Soft Material Service of the Institut de Ciència de Materials de Barcelona (ICMAB-CSIC, Bellaterra, Catalonia, Spain). Samples were previously diluted to a concentration of approximately 10^8^ particles/mL. Sample injection was performed using a pump to improve the robustness of the measurement by continuous addition, and to minimize the photobleaching effect due to fluorescence depletion over time. 60 s videos were recorded at RT and analyzed with the NTA 3.4 software (Malvern Panalytical, Malvern, UK). Tracked particle size was determined from its Brownian motion. Three independent experimental replicates were carried out for each sample. Camera level and detection threshold were manually adjusted for each replica.

### 2.8. Total Protein and Spike Quantifications

A BCA Protein Assay (#23225, Thermo Fisher Scientific, Waltham, MA, USA) was performed following manufacturer’s instructions using the provided BSA as standard. Colorimetric absorbance at 562 nm was read on a Multilabel Plate Reader Victor3 (Perkin Elmer, Waltham, MA, USA).

For SARS-CoV-2 Spike quantification, samples were charged into Bio-Dot Apparatus (#1706545, Bio-Rad, Hercules, CA, USA) while a low vacuum was applied. Nitrocellulose membrane (#88018, Thermo Fisher Scientific, Waltham, MA, USA) was placed at the top of humidified filter paper. Once samples were transferred, membrane was incubated with anti-SARS-CoV-2 spike glyco-protein S2 monoclonal antibody (Ab281312, AbCam, Cambridge, UK) and an anti-rabbit secondary antibody (A9919, Merck, Kenilworth, NJ, USA) following the same procedure previously published for Western blot [12]. Once dried, membranes were scanned, and the pixel density for each loaded sample was analyzed using software ImageJ2 Fiji 2.9.0 (National Institutes of Health, Bethesda, MD, USA). The standard used for quantification was a recombinant human coronavirus SARS-CoV-2 spike glycoprotein S2 subunit (Ab272106, AbCam, Cambridge, UK).

### 2.9. Human Sera Assay

The sera used in this study were provided by the Biobank of the Banc de Sang i Teixits (BST) and samples were anonymized. Eight samples from convalescent non-vaccinated COVID-19 patients (confirmed with RT-qPCR) were used in this work. Four sera collected from COVID-19-uninfected and -unvaccinated individuals were used as negative control.

Briefly, VLP variants and controls were charged into Accutran-Cross Blot-System for Cross Blot (#448100, Schleicher&Schuell, Dassel, Germany) containing vertical lane-shaped wells above an immobilized nitrocellulose membrane (#88018, Thermo Fisher Scientific, Waltham, MA, USA) and incubated overnight at 4 °C with agitation. Once antigens were transferred, membranes were incubated for 1 h in agitation with blocking buffer (2% (*w*/*v*) nonfat dry milk in 1× PBS). After blocking, membranes were incubated with human sera (1:60 dilutions in blocking buffer) for 2 h, charged in horizontal lane-shaped wells. After sera incubation, membranes were incubated with an anti-Human IgG (Fab specific)-peroxidase antibody produced in goat (#A0293, Sigma Aldrich, St. Louis, MO, USA) (1:2000 in blocking buffer) for 1 h. Then, they were revealed with Pierce™ ECL Plus western blotting substrate (32132, Thermo Fisher Scientific, Waltham, MA, USA). Once dried, membranes were imaged using a ChemiDoc™ Touch Imaging System (#1708370, Bio-Rad, Hercules, CA, USA) and pixel densities were analyzed using the software ImageJ2 Fiji (National Institutes of Health, Bethesda, MD, USA). All the wash steps between incubations were performed with agitation in 0.05% Tween-20 in 1× PBS. The antigen used as positive control was a SARS-CoV-2 Spike S1-His Recombinant Protein (#40591-V08H, SinoBiological Europe GmbH, Eschborn, Germany).

## 3. Results

### 3.1. Engineered Spike Protein Variants

**Figure 2:**
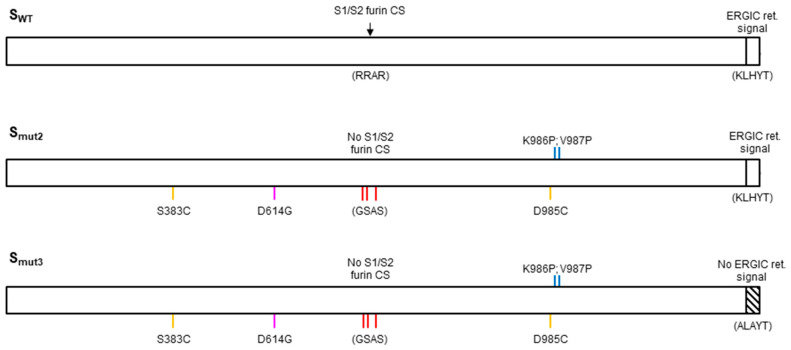
Schematic representation of the S_WT_ protein and the S_mut2_ and S_mut3_ variants with its mutations indicated. Furin cleavage site (CS) is indicated by an arrow. ER-Golgi intermediate compartment retention signal (ERGIC ret. signal) is represented as a white box.

Three S protein variants were generated and studied, named S_WT_, S_mut2_ and S_mut3_. S_WT_ codes for the original Wuhan sequence. S_mut2_ incorporates two proline substitutions, D614G as the early dominant pandemic form, the elimination of the S1/S2 cleavage site and the creation of a disulfide bond to avoid ADE, by substitution of two cysteines (Figure 2). S_mut3_ incorporates the same mutations as S_mut2_ with additional substitutions in its C-terminus in order to eliminate its ER-Golgi intermediate compartment (ERGIC) retention motif (Figure 2).

#### 3.1.1. Proline Substitutions

The spike protein can transition between an unstable prefusion state to a postfusion stable conformation, as a consequence of its role in membrane fusion [25]. The protein structural design generated with K986P and V987P proline substitutions is used and described as a strategy that can be applied in different Betacoronavirus S proteins, for stabilizing and retaining them in the antigenically optimal prefusion conformation by inactivating its membrane fusion activity [24,33]. This approach improves its expression yields and conformational homogeneity [24,34]. Prefusion-stabilized antibody epitopes are more likely to lead to neutralizing antibody responses, and it has been demonstrated that these mutations were able to elicit high neutralizing antibody titers against MERS-CoV [24,25,33]. Indeed, several SARS-CoV-2 vaccines incorporate stabilizing proline substitutions for the mentioned reasons [35,36].

#### 3.1.2. Cysteine Substitutions

Antibodies are generally beneficial and protective against viral infections [29]. However, in SARS-CoV-2 infection, sub-optimal antibody production and early seroconversion has been reported to correlate with disease severity by ADE phenomenon, which can occur mediated by the engagement of Fc receptors (expressed on monocytes, macrophages, and B cells among others) [29,37]. This phenomenon may promote ACE-2-independent viral entry to cells expressing Fc receptors (FcRs) [38]. As the quality and quantity of the antibody is crucial in order to elicit a good and effective immune protection against SARS-CoV-2, the literature suggests that the disulfide bond created by cysteine substitutions S383C and D985C will hide some of the SARS-CoV-2 S immunogen non-neutralizing epitopes that might cause an ADE response [22,39]. Although low expression yields have been reported when expressing S proteins harboring S383C and D985C mutations, stabilizing mutations like proline substitutions may increase their immunogenic potential [22,40].

#### 3.1.3. Early Dominant Pandemic Form

Despite not presenting alarmingly high mutation ratios, during the COVID-19 pandemic, SARS-CoV-2 experienced different sequence variations, which resulted in changes on its transmissibility, severity, and immune escape [20,41]. The D614G variant, presenting a glycine (G) substitution of the aspartic (D) present at the 614 position of the original S sequence, was rare in February 2020 [26]. However, it quickly replaced the ancestral virus, becoming the dominant pandemic form worldwide by April 2020 [42]. The D614G mutation resulted in a fitness advantage without increased severity, appearing to correlate with higher viral loads in patients and increasing in vitro infectivity [26,43]. It has been suggested that the higher infectivity of the G614 variant is mostly caused by its increased stability when forming S trimers, preventing its premature loss, and hence effectively increasing the number of S proteins that can facilitate the infection [42]. Studies have shown that G614 has a higher neutralization sensitivity to COVID-19 convalescent human sera when compared to the ancestral variant, suggesting an increased epitope exposure [44]. Its conformational changes, improving stability, preventing premature loss, and favoring prefusion conformations, make D614G an interesting mutation to incorporate for the generation of new vaccine candidates [42].

#### 3.1.4. Furin Cleavage Site Removal

As suggested in different studies, probably all coronavirus S proteins are cleaved at some point during infection, and in many cases, this cleavage occurs at the S1/S2 position [27]. The SARS-CoV-2 S protein sequence contains a S1/S2 polybasic cleavage site (CS), which can be recognized and cleaved by host cell furin protease [45]. Upon S1/S2 cleavage, a second CS present at the S2 domain becomes exposed, which, after further cleavage, activates the S2 membrane fusion machinery [45]. It has been demonstrated that the S1/S2 CS increases SARS-CoV-2 pathogenesis [46,47] and promotes its entry into lung cells [45,48]. However, studies on prefusion-stabilized S immunogens presenting proline substitutions (Section 3.1.1) concluded that no large conformational changes were imparted as a consequence of the furin S1/S2 cleavage absence [25]. This justifies its removal in order to generate more homogeneous vaccine immunogens [25]. To do so, the 682–685 RRAR sequence of the S protein was substituted by GSAS [22].

#### 3.1.5. Dibasic Motif Removal

SARS-CoV-2 protein presents a dibasic motif (KXHXX) in the last four amino acids of its cytoplasmic tail, which specifies intracellular localization [49]. This motif reduces the S rate of traffic through the Golgi complex and promotes its retention at ERGIC [50,51]. Studies showed that when lysine (K) and histidine (H) residues present in the dibasic motif were substituted by alanines, efficient transport and localization at the plasmatic membrane occurred [28,50,51]. S-VLPs are generated by the incorporation of the S protein present at the cell membrane into the budding Gag-based particles. For that reason, the removal of the ERGIC retention signal was hypothesized to favor the generation of S-VLPs.

### 3.2. S-VLPs Variant Production and Quantification

To generate the S-VLP candidates, exponentially growing HEK293 cells in 1 L suspension Erlenmeyer flasks were transfected with PEI as transfection reagent according to Table 1. Negative control VLPs produced by the co-transfection of Gag::eGFP and an empty plasmid (G-VLPs) were also generated to quantify and subtract the unspecific binding of the polyclonal human sera to the non-functionalized VLP scaffold. The HIV-1 Gag polyprotein used in this work was fused in frame with the eGFP reporter, in order to facilitate tracking and quantification of the produced VLPs [15].

**Figure 3:**
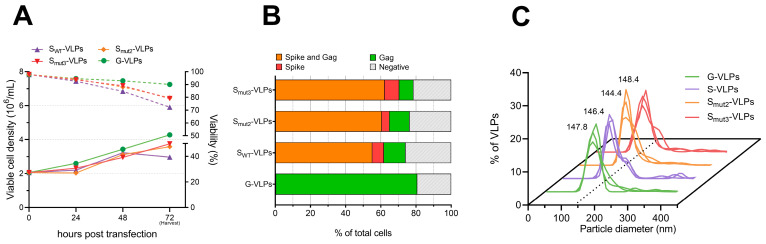
(**A**): Cell concentrations (solid lines) and viabilities (dotted lines) of HEK293 cells transfected for the production of different VLP variants, described in Table 1. (**B**): Transfected populations, analyzed by ICC at 72 hpt. (**C**): Particle size distribution of the purified S VLP variants, analyzed by nanoparticle tracking analysis (NTA), with the mode diameter of each variant indicated.

Cells transfected with Gag and S proteins showed viabilities between 70–80% at harvest time (Figure 3A). Interestingly, cells transfected with S_mut2_ and S_mut3_ variants showed a ~10% viability improvement compared to S_WT_ expression, reaching higher cellular densities as well. This may be favored by the stabilizing mutations, which facilitate protein expression. No significant differences in terms of viability and cell growth were observed between the S_mut2_ and S_mut3_ variants, indicating that the suppression of the ERGIC retention motif does not have any effect on this aspect.

Additionally, ICC analysis showed that the expression of the mutated S variants was translated into an increase in the total populations expressing S proteins and the double-positive population co-expressing Gag and S, which rose from 55.2% for the S_WT_ VLP variant to 60.6% and 62.2% for S_mut2_ and S_mut3_ VLP variants, respectively (Figure 3B). Concordantly, confocal microscopy images appeared to exhibit a higher intensity of the S protein signal and Gag co-localization on the cellular membrane for the S_mut2_ and especially the S_mut3_ variants when compared to S_WT_ producer cells (Supplementary Materials Figure S1).

VLPs were purified from the harvested supernatants by sucrose cushion ultracentrifugation. After purification, the group transfected with the unmodified S_WT_ protein showed VLP concentrations of 2.9 × 10^10^ particles/mL, compared with 6.38 × 10^10^ and 4.69 × 10^10^ VLPs/mL for S_mut2_ and S_mut3_ VLP variants, respectively, representing 2.2- and 1.6-fold increases. No significant morphological differences were detected under transmission electron microscopy (TEM) between groups (Supplementary Materials Figure S3). Particle size analysis demonstrated high diameter uniformity among the produced VLPs, and revealed that there were no statistically significant differences in particle size distribution between all the produced VLPs (Figure 3C). The mode diameters were also nearly identical for the control and the S VLP variants, ranging from 144.4 to 148.4 nm (Figure 3C). These findings indicate a high degree of uniformity in particle size across all the generated VLP variants. Additionally, the VLP ratio in respect to total extracellular particles improved from 8.3% for the S_WT_-VLPs to 16.2% and 14% for the S_mut2_ and S_mut3_ VLPs (Table 2), suggesting a positive effect of the mutations harbored by the two mutants enhancing the VLP production and improving its ratio among the total extracellular particles.

The total protein concentration of the purified Smut2 and Smut3 VLPs was approximately 50% of that for SWT group (Table 2), although presenting a higher VLP concentration. Considering that functionalized VLP variants presented higher VLP concentrations, this seems to indicate that the VLPs generated by the expression of the Smut2 Smut3 variants present less undesired host cell proteins. A deeper VLP characterization including proteomic analysis could help to better define the host cell protein composition of the produced particles to explain the observed phenomena [52].

Dot blot analysis allowed to determine the spike concentration of each VLP purified candidate. The S protein concentration was similar for the purified Smut2-VLPs and SWT-VLPs. Interestingly, it was increased 2.2-fold when expressing the Smut3 variant, suggesting a positive effect of the removal of the ERGIC retention motif favoring the S localization at the plasmatic membrane and subsequently facilitating its incorporation to the produced VLPs. This was translated in a greater functionalization of the Smut3-VLPs, with ~2460 spike proteins per VLP (Table 2).

### 3.3. Assessment of the Immunogenic Potential of the S-VLP Candidates

To assess the immunogenic potential of the generated SWT, Smut2, and Smut3 VLPs, they were tested for their recognition by human convalescent COVID-19 patient sera. G-VLPs were used as negative control and a recombinant commercial S protein was used as a positive control. A total of 900 ng (total protein) of each VLP candidate was transferred into a nitrocellulose membrane and treated with different patient sera, as detailed in Section 2.7.

Previously, the level of antibodies against SARS-CoV-2 for each sera sample was typified to determine each patient’s immune response level (Supplementary Materials Table S1).

**Figure 4:**
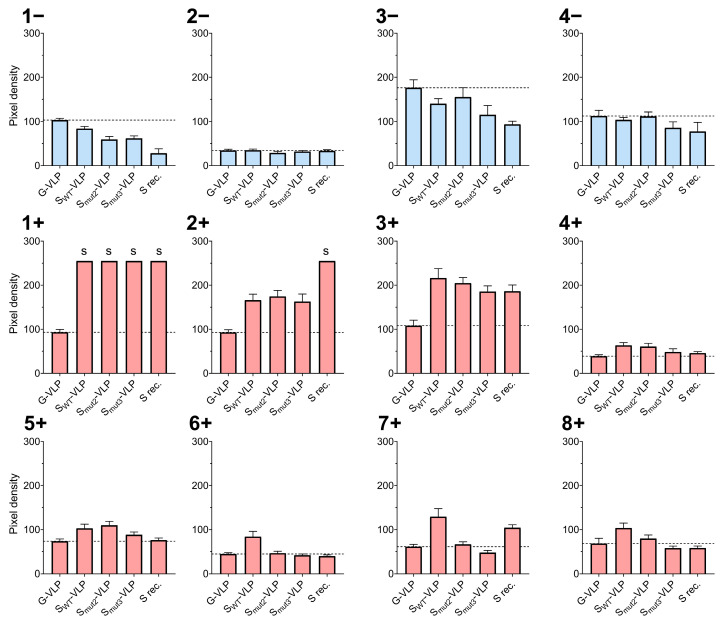
Relative pixel densities of the VLP recognition assays by negative (blue) and positive (red) tested sera samples. Horizontal dashed line indicates the unspecific recognition threshold, marked by the pixel density of the G-VLP negative control. Saturated pixel densities are indicated with an “s”. Soluble spike recombinant protein (S rec.) was used as positive control.

The analysis of the pixel density of the membrane allowed for the quantification of the antigen recognition by each individual serum. The unspecific signal against the control G-VLPs was used to determine the S-specific immune response threshold for each serum (dashed lines, Figure 4).

As expected, negative sera did not present S-specific antibody recognition of the S-VLP variants nor the S positive control (Figure 4). Generally, all the COVID-19 convalescent sera showed specific immune responses against S-VLP variants greater than its unspecific G-VLP threshold. Patients coded as 1+, 2+, and 3+ presented the highest antigen recognitions in agreement with their previously determined high anti-SARS-CoV-2 antibody positivity (Supplementary Materials Table S1). Particularly, 1+ reached the pixel saturation limit for all the studied S immunogens (not allowing for their comparative study), whereas sera 2+ only showed pixel saturation for the S recombinant positive control, which was significantly more recognized than the S-VLPs (Figure 4). Interestingly, and except for sera 1+ and 2+, S-VLPs were generally better recognized by the convalescent sera than the recombinant S protein control. This can be explained as the S-VLPs present the S protein in a more genuine conformation than the individual soluble S protein. Overall, the serum assays better recognized the SWT-VLP immunogens than the Smut2 and Smut3 VLP variants (Figure 4). This was not the case for sera 2+ and 5+, although they did not show large significant differences between SWT and Smut2 VLP recognition. Additionally, and contrarily to what might be expected given the large number of S protein units presented on its surface, Smut3-VLPs presented the worst recognition levels by all the tested convalescent sera (Figure 4). The obtained results made us conclude that the unmodified wild-type S protein was the best candidate for the functionalization of Gag-based VLPs for their use as immunogens.

## 4. Discussion

The current VLP-based SARS-CoV-2 vaccine candidates in clinical development face various obstacles, such as limited production efficiency, sub-optimal spike functionalization, differences in glycosylation depending on the selected expression platform, and complex upstream and downstream processing demands [53]. In this work, the expressions of three different rationally engineered VLP candidates were studied. Those candidates, named S_WT_-VLP, S_mut2_-VLP, and S_mut3_-VLP, were generated by the co-expression of the Gag::eGFP and a spike protein variant: S_WT_, S_mut2_, and S_mut3_, respectively. For this purpose, we adopted a highly immunogenic [54] co-transfection approach established in our previous research for the production of S VLPs, which successfully confirmed incorporation of the Spike protein into the VLPs [12]. This method was not only effective but also demonstrated scalability up to a 1 L bioreactor scale, combined with a scalable purification process, ensuring its potential for large-scale application while facilitating adoption to cGMP standards [12].

Cells expressing the two mutated S variants behaved similarly, displaying approximately a 10% viability improvement and an increased cell density at harvest time (72 hpt) compared with the S_WT_ group, which had viabilities of ~72%. Such behavior could be a consequence of the stabilizing mutations introduced to the mutated spike proteins facilitating its expression [24,40]. The obtained viabilities are concordant with the values previously observed in PEI-mediated transfection for the production of HIV-1 Gag-based VLPs, typically within the range of 70–80% viable cells at the time of harvest [12,55,56].

The reproducibility of the presented co-transfection approach becomes evident when we compare the ICC analysis of the double-positive expresser populations for the S_WT_-VLP expresser cells (55.2%) with the previous research, where the double-transfected population accounted for 55.1% [12]. The results presented in this study demonstrated increases of up to 60.6% and 62.2% of double-positive expression populations for S_mut2_ and S_mut3_ groups, a desirable outcome, denoting that a higher proportion of the cultured cells are generating functionalized Gag VLPs.

After purification, S_mut2_ and S_mut3_ candidates presented 2.2- and 1.6-fold VLP concentration increases compared with S_WT_-VLPs. Additionally, the VLP ratio among total extracellular particles for the S_mut2_ and S_mut3_ VLPs improved to 16.2% and 14% when compared with the 8.3% obtained for the S_WT_-VLPs. Dot blot analysis of the purified VLP candidates allowed one to determine that S_mut3_-VLPs presented a 2.2-fold increase in S protein concentration compared to the S_WT_-VLPs and S_mut2_-VLPs, and greater Spike functionalization per VLP. Establishing the specific effects of various mutations at the same time can be challenging, as each mutation may have different impacts on protein expression, especially when combined. However, the results from studying transfection (culture growth, expresser populations, confocal microscopy analysis) and characterizing the generated VLPs (concentration, ratio over total particles, S protein concentration, and VLP functionalization) provide evidence that these introduced mutations contributed positively to the generation of VLPs without significantly altering their particle size distributions or mode diameters. Notably, the deletion of the ERGIC retention motif appears to have a particular role in the functionalization of VLPs with the S protein, as S_mut3_ presented remarkably greater functionalization levels (Spike ratio per VLP) than its counterpart variant S_mut2_. Although previous studies have analyzed the trafficking itinerary of the S protein, both by itself [57], and in combination with E and M SARS-CoV-2 proteins [58], future studies should be conducted to examine the subcellular S protein localization, processing, and transport to the plasma membrane when co-expressed with HIV-1 Gag polyprotein; to fully understand the role that the ERGIC motif plays on the generation of Gag-based Spike VLPs. 

Further, the obtained VLP immunogens were tested for their recognition by COVID-19 convalescent human sera to evaluate their immunogenicity and potential efficacy as vaccine candidates. All the different COVID-19 patients’ convalescent sera recognized the VLP variants above the G-VLP threshold, varying on intensity depending on their antibody positivity against SARS-CoV-2. This is concordant with the literature, where a wide spectrum of antibody responses against Spike VLPs in COVID-19 convalescent sera can be observed, ranging from low to very high levels, probably influenced by the severity and time since the infection [34]. In general, S-VLPs were better recognized by the convalescent sera compared with the soluble version of the S protein, due to the advantageous nature of the VLPs when it comes to proper antigen presentation in terms of conformation and protein context [10,11]. Surprisingly, the assay determined that most of the sera showed the best recognition levels for the unmodified S_WT_-VLPs. In contrast, and contrarily to what might be expected given its S functionalization levels, S_mut3_-VLPs presented the worst convalescent sera recognition levels.

This work concludes that although improving the expression of VLPs and their functionalization with the S immunogen, the presented S_mut2_ and S_mut3_ VLP candidates did not show immune responses improving or equaling the conventional S_WT_-VLPs. Further studies need to be performed to fully understand the immunogenic potential of the presented VLP variants, as the low recognition by convalescent sera could be in part due to the concealment of some regions containing epitopes with the potential to trigger undesired ADE responses [37,59]. Moreover, conducting a more extensive study of S VLP recognition by a larger pool of patients’ sera could provide additional and valuable insights. Such an expanded study would help to better understand S VLP recognition, particularly across diverse demographic strata, including distinctions related to age, gender, or medical history [60].

The thermostability and aggregation of Gag-based VLPs had been previously studied, concluding that they can be stable up to three months at 4 or −80 °C [14]. However, additional tests on the S VLP variants presented in this work need to be conducted, as mutations such as proline substitutions could affect the ability of the proteins to withstand heat stress or freeze–thaw cycles [40]. This is particularly important because instability associated with cold chain storage during clinical development and commercial distribution is a critical challenge that can significantly impact the efficiency of a vaccine candidate [61].

Finally, future research should focus on the ability of the presented candidates to elicit strong protective cellular and humoral immune responses in immunized mice, and compare the outcomes with those reported in the current literature, particularly for Spike VLPs based on MLV-Gag viral proteins [34] and Spike VLPs based on M and E SARS-CoV-2 proteins [53]. Such studies will help to determine if the high flexibility and strong immunogenicity of Gag-based VLPs [54] make them a better carrier platform for the presentation of the Spike protein to the vaccinated individuals.

## Figures and Tables

**Table 1:** Summary of the tested VLP candidates, its mutations, and plasmids used to generate them.

VLP	Spike Mutations	Plasmids Transfected
S_WT_-VLP	Original Wuhan-Hu-1 spike glycoprotein sequence	pSpike + pGag::eGFP
S_mut2_-VLP	K986P, V987P, S383C, D985C, D614G, R682_R685delinsGSAS	pSpikemut2 + pGag::eGFP
S_mut3_-VLP	K986P, V987P, S383C, D985C, D614G, R682_R685delinsGSAS, K1269A H1271A	pSpikemut3 + pGag::eGFP
G-VLP	-	pGag::eGFP + pMock

**Table 2:** Characteristics of each produced S-VLP candidate and control (G-VLPs).

	VLPs/mL	Total Particles/mL	VLP/TP (%)	Total Protein Conc. (µg/mL)	Spike Conc. (µg/mL)	Spike/VLP * (Units/VLP)
S_WT_-VLPs	(2.90 ± 0.13) × 10^10^	(3.51 ± 0.13) × 10^11^	8.3	566.14 ± 19	12.08 ± 0.99	1776.8
S_mut2_-VLPs	(6.38 ± 0.05) × 10^10^	(3.94 ± 0.2) × 10^11^	16.2	263.99 ± 15	13.90 ± 0.35	929.3
S_mut3_-VLPs	(4.69 ± 0.05) × 10^10^	(3.35 ± 0.07) × 10^11^	14.0	297.88 ± 104	27.05 ± 4.03	2460.2
G-VLPs	(1.85 ± 0.04) × 10^11^	(5.15 ± 0.24) × 10^11^	35.9	305.83 ± 46	-	-

* Assuming a Mw of 141.178 kDa for the Spike protein.

## Data Availability

The data presented in this study are available on request from the corresponding author.

## Supplementary Materials

The following supporting information can be downloaded at: https://www.mdpi.com/article/10.3390/vaccines11111641/s1, Figure S1: Confocal microscopy images; Figure S2: Spike Dot Blot uncropped membrane; Figure S3. Transmission Electron Microscopy (TEM) of the purified VLPs; Table S1: Summary of positive and negative sera samples.
