# Supplementary material for: Gag Virus-like Particles Functionalized with SARS-CoV-2 Variants: Generation, Characterization and Recognition by COVID-19 Convalescent Patients’ Sera
Source: Vaccines (Basel). 2023 Oct 26;11(11):1641. doi: 10.3390/vaccines11111641 (PMC10675557; doi:10.3390/vaccines11111641)
Supplement: Supplementary file 1 [file vaccines-11-01641-s001.zip › vaccines-2634670-supplementary.pdf]

Figure S1. Confocal microscopy images. Spike (red), Gag (green), nuclei (blue)

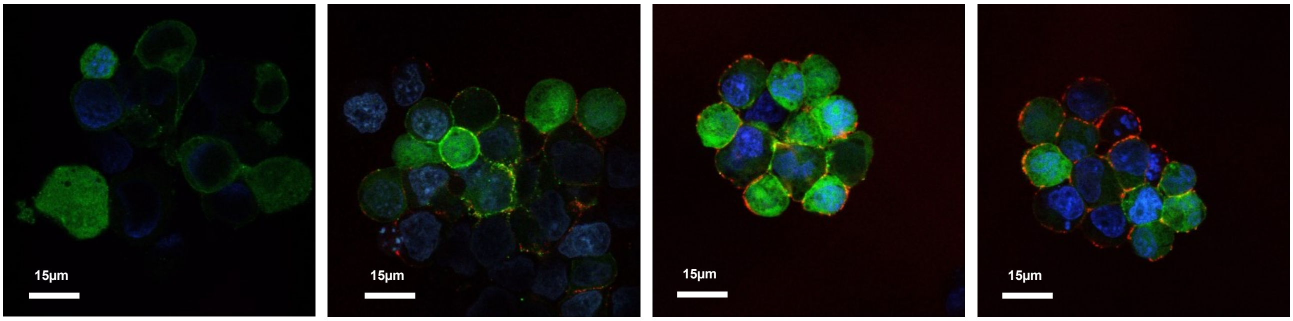

Figure S2. Spike Dot Blot uncropped membrane

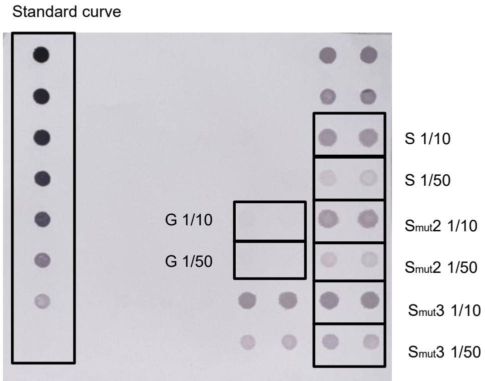

Figure S3. Transmission Electron Microscopy (TEM) of the purified VLPs.

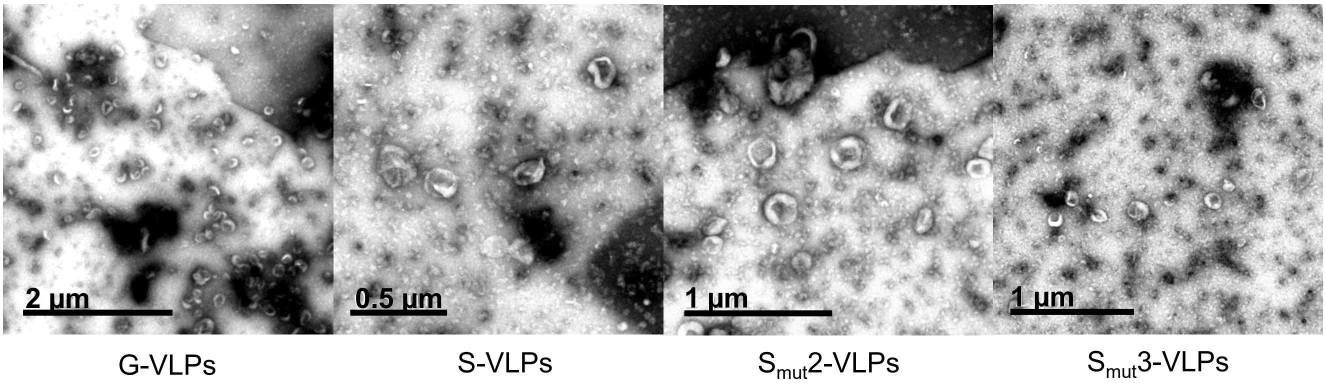

Table S1. Summary of positive and negative sera samples. Their antibody levels against SARS-CoV-2 are indicated in arbitrary units (AU) per mL.

Code	Antibody positivity against SARS-CoV-2 (AU/mL)	Code	Antibody positivity against SARS-CoV-2 (AU/mL)
1-	2.5 (negative)	1+	4046.4
2-	8.1 (negative)	2+	307.5
3-	1.1 (negative)	3+	214.21
4-	0.1 (negative)	4+	98.6
		5+	105.7
		6+	135.1
		7+	427.3
		8+	132.8
